# Pilus Operon Evolution in *Streptococcus pneumoniae* Is Driven by Positive Selection and Recombination

**DOI:** 10.1371/journal.pone.0003660

**Published:** 2008-11-06

**Authors:** Alessandro Muzzi, Monica Moschioni, Antonello Covacci, Rino Rappuoli, Claudio Donati

**Affiliations:** Novartis Vaccines and Diagnostics, Siena, Italy; University of British Columbia, Canada

## Abstract

**Background:**

The evolution of bacterial organelles involved in host-pathogen interactions is subject to intense and competing selective pressures due to the need to maintain function while escaping the host immune response. To characterize the interplay of these forces in an important pathogen, we sequenced the *rlrA* islet, a chromosomal region encoding for a pilus-like structure involved in adherence to lung epithelial cells in vitro and in colonization in a murine model of infection, in 44 clinical isolates of *Streptococcus pneumoniae*.

**Results:**

We found that the *rrgA* and *rrgB* genes, encoding the main structural components of the pilus, are under the action of positive selection. In contrast, the *rrgC* gene, coding for a component present in low quantities in the assembled pilus, and the *srtB*, *srtC* and *srtD* genes, coding for three sortase enzymes essential for pilus assembly but probably not directly exposed to the host immune system, show no evidence of positive selection. We found several events of homologous recombination in the region containing these genes, identifying 4 major recombination hotspots. An analysis of the most recent recombination events shows a high level of mosaicism of the region coding for the *rrgC*, *srtB*, *srtC* and *srtD* genes.

**Conclusions:**

In the *rlrA* islet, the genes coding for proteins directly exposed to the host immune response are under the action of positive selection, and exist in distinct forms in the population of circulating strains. The genes coding for proteins not directly exposed on the surface of the bacterial cell are more conserved probably due to the homogenizing effect of recombination.

## Introduction

*Streptococcus pneumoniae* has recently been shown to express a pilus-like structure that is involved in adherence to lung epithelial cells in vitro, as well as in colonization in a murine model of infection [1]–[4]. These structures are encoded in the *rlrA* islet [1], [5], a chromosomal region of approximately 11 kb, containing seven genes: *rlrA* coding for a *rofA*-like transcriptional regulator, *rrgA*, *rrgB* and *rrgC* coding for three LPXTG cell-wall anchored proteins, and *srtB*, *srtC* and *srtD*, coding for three specific sortase enzymes. The *rlrA* islet is present in a subset of the circulating strains [6], with an high degree of correlation with the clonal complexes defined by Multi Locus Sequence Typing (MLST) [7], indicating that the *rlrA* islet is inherited clonally and is stable on the evolutionary time-scale described by MLST. Based on sequence conservation, the strains that encode the *rlrA* islet can be grouped into three major clades that also correlate with the MLST clonal complexes [6]. Recently, a second independent pilus islet has been identified in *S. pneumoniae* [8], which is also present in a subset of the circulating strains and which in some strains coexist with *rlrA*. This second islet, although sharing the general organization of pilus-encoding islets in gram-positive bacteria [9], is composed by a different number of genes and is only remotely related to *rlrA*.

In order to analyze the processes that influence the variability of the *rlrA* islet in the population of circulating strains, we report the nucleotide sequences of this locus in 44 clinical isolates of *S. pneumoniae*. Previous studies have pointed out the importance of recombination in the molecular evolution of *S. pneumoniae* [10], and molecular phylogenetic data of 7 housekeeping genes have allowed to estimate that in *S. pneumoniae* the probability that an allele changes by recombination is 8.9 times higher than the probability that it changes by mutation [11]. Recent recombination events can be detected in sequence data by looking at polymorphic sites and performing the so-called four gametes test, that, in the absence of homoplasy, identifies recombination by the occurrence all four possible two-locus haplotypes in pairs of biallelic single nucleotide polymorphisms. Alternatively, recombination can be inferred from differences in the phylogenetic trees computed from different regions of the sequences. However, only a small portion of the recombination events can be identified using similar model–free methods, the main limiting factors being the genetic diversity of the region and the age of the event. Recently, model-based methods to analyze population genetics data explicitly allowing for variable recombination rates have been developed, and have been able to identify regions where recombination is more likely to occur (hotspots) and to measure site-specific recombination rates [12], [13]. Although computationally expensive, these methods provide a more realistic estimate of the amount of recombination present in a population genetics data set [14].

In pathogens, the evolution of surface exposed proteins is subject to the additional constraint that they must avoid reconnaissance from the host immune system, and therefore they often display an increased level of sequence variability if compared to proteins performing core metabolic functions. Population genetics studies have evidenced for this class of proteins the role of positive selection, a mechanism that causes an increased rate of fixation of new alleles due to periodic selective sweeps [15]–[23]. Genes undergoing positive selection are identified from sequence data by measuring the ratio of non-synonymous to synonymous substitutions, *d_N_/d_S_*. If selection has no effect on fitness, both types of mutations are fixed at the same rate and *d_N_/d_S_* = 1, while in the case of positive selection, non-synonymous substitutions are fixed at a rate exceeding that of synonymous (or silent) substitutions, and the ratio *d_N_/d_S_* is larger than 1. Since it is unlikely that selection acts evenly on all sequence positions, measuring *d_N_/d_S_* averaged over the length of entire genes only rarely detects positive selection. This difficulty is overcome by likelihood-based methods, allowing the estimate of distinct *d_N_/d_S_* ratios for each codon [24]–[26]. In these approaches, codons are partitioned amongst classes with different values of *d_N_/d_S_* by maximizing the likelihood of the observed sequences, and the results are contrasted against the null hypothesis that no site has *d_N_/d_S_*>1 by a likelihood ratio test. Although estimates of the presence of positive selection based on these tests are known to be conservative, sites under positive selection are then identified from the high posterior probabilities of belonging to classes with *d_N_/d_S_*>1.

The *rlrA* islet is an ideal test case to study the molecular evolution of the different components of bacterial organelles involved in interactions with the host and to identify the different effects of mutation and recombination in the genomic regions coding for these structures, since it contains both the genes (*rrgA*, *rrgB*, and *rrgC*) that code for the structural components of the pilus [1], [2], [4], and the genes (*srtB*, *srtC* and *srtD*) that code for the sortase enzymes that are essential for pilus assembly [1], but are unlikely to be exposed to the host immune system. We found in the *rlrA* islet a complex pattern of regions under positive selection, where recombination has played only a minor role, and regions where recombination appears to have been the major evolutionary mechanism. The regions under positive selection coincide with the *rrgA* and *rrgB* genes, which are the most variable genes in the *rlrA* islet. For the *rrgC*, *srtB*, *srtC* and *srtD* genes, that show a much lower level of diversification compared to *rrgA* and *rrgB*, we found no evidence of positive selection. In the region encoding these genes we identified several recombination events that may have contributed to decrease their sequence diversity compared to the *rrgA* and *rrgB* genes, and a map of the recombination rate across the *rlrA* islet showed the presence of 4 major recombination hotspots.

## Results

The *rlrA* islets from 44 strains of *S. pneumoniae* were sequenced, and additionally the nucleotide sequence of the *rlrA* islet was extracted from 4 complete genomes downloaded from the NCBI and TIGR web site. The selected strains (see Text S1 and Table S1) are highly representative of the variability of *S. pneumoniae*, in term of Serotype, MLST Sequence Type, geographic origin and associated disease.

### Sequence variability

**Figure 1:**
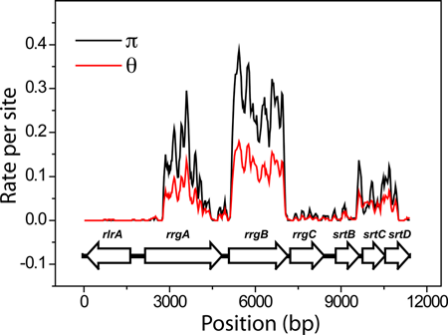
Genetic diversity of the pilus operon. Nucleotide diversity π and Watterson estimator θ of the population mutation rate per site measured along the sequence. The positions of the ORFs on the sequence are also shown.

The nucleotide sequences of the *rlrA* islet were aligned, and the nucleotide diversity π and the Watterson estimator θ of the population mutation rate per site were computed using a sliding window of 100 bp along the sequences. The results are reported in Fig. 1, where the positions of the genes are also shown. In Table 1 we also report the average values of π and θ for the 7 genes contained in the *rlrA* islet. Both π and θ have two peaks that correspond remarkably well with the *rrgA* and *rrgB* genes, which code for two of the three structural components of the pilus [1]. The next area where π and θ are positive is the one containing the genes encoding the sortase enzymes. However, in this region both π and θ have values much lower than those in the *rrgA* and *rrgB* genes. Remarkably, in the *rrgC* gene, that codes for one structural components which is present in low abundance in the assembled pilus [2], both π and θ have values very close to 0. The *rlrA* regulator is the most conserved gene, having only 3 segregating sites and 4 distinct haplotypes.

**Table 1:** Population genetics data.

Gene name	*rlrA*	*rrgA*	*rrgB*	*rrgC*	*srtB*	*srtC*	*srtD*
Length (bp)	1527	2685	2052	1179	831	790	849
Haplotypes	4	18	18	10	7	9	7
Segregating sites	3	428	960	23	40	126	87
θ	0.00011	0.04	0.162	0.004	0.011	0.040	0.024
π	0.00011	0.084	0.335	0.008	0.019	0.048	0.042
π synonymous	0.00024	0.204	0.608	0.016	0.061	0.163	0.121
π nonsynonymous	0.00007	0.054	0.277	0.006	0.007	0.020	0.021

Length, number of haplotypes, number of segregating sites, Watterson θ, pair-wise sequence diversity π, π for synonymous sites and π for non-synonymous sites for the 7 genes in the *rlrA* islet.

### Positive selection

A possible explanation of the differences in sequence variability amongst the genes forming the *rlrA* islet could reside in the different selective pressure exerted on them by the host immune response. To confirm this hypothesis, and to distinguish the possibility that the host immune system produces a positive selective pressure from a relaxation of the selective constraints, we estimated the non-synonymous to synonymous substitution ratio (*d_N_/d_S_*) for all the genes in the *rlrA* locus using a codon-based Maximum Likelihood method [24]. In these tests, the likelihood of a substitution model where the rate of non-synonymous substitutions for all codons is constrained to be equal or lower than the rate of synonymous substitutions (*d_N_/d_S_*≤1) is compared to the likelihood that some sites are allowed to have *d_N_/d_S_*>1. The latter models incorporate the possibility of positive selection, while the former models constrain the evolution of all codons to be purifying (*d_N_/d_S_*<1) or nearly neutral (*d_N_/d_S_*≈1). If the high level of divergence of the sequences is due only to a relaxation of the selective constraints on the encoded proteins, incorporating *d_N_/d_S_*>1 does not provide a significant higher likelihood than models where all codons are forced to have *d_N_/d_S_*≤1. Conversely, a significantly higher likelihood of models incorporating *d_N_/d_S_*>1 indicates that relaxed selective constraints alone cannot explain the high level of sequence diversity for some of the genes in the *rlrA* islet. The results of a likelihood ratio test between I) the M1a (nearly neutral evolution) and M2a (positive selection) models, and II) the M7(β) (nearly neutral evolution) and M8(β+ω_s_>1) (positive selection) models implemented by the *codeml* program from the PAML package [24] are shown in Table 2.

**Table 2:** Tests of positive selection.

Gene	M0	M1a	M2a	M2a-M1a	χ^2^_2_( M2a-M1a)	M7	M8	M8-M7	χ^2^_2_( M8-M7)
*rrgA*	−5314.2676	−5247.41	−5236.66	10.75	**2.15E-05**	−5248.30	−5236.67	11.63	**8.87E-06**
*rrgB*	−6047.8218	−5936.92	−5935.86	1.06	0.34	−5930.65	−5925.23	5.41	**4.46E-03**
*rrgC*	−1740.9345	−1740.21	−1740.01	0.20	0.82	−1740.25	−1740.01	0.24	0.78
*srtB*	−1312.8999	−1312.45	−1312.35	0.10	0.90	−1312.47	−1312.47	−1.6E-04	1.00
*srtC*	−1618.7204	−1617.31	−1617.31	0.00	1.00	−1617.12	−1617.12	−3.4E-05	1.00
*srtD*	−1502.4508	−1492.71	−1489.88	2.82	0.06	−1492.88	−1489.89	2.99	0.05

In this table we report the log-likelihood data and the associated χ^2^_2_ for the evolutionary models tested with PAML [24]. P-values below 0.01 are marked in bold. Only in the case of *rrgA* both the M2a-M1a (test I) and M8-M7 (test II) Likelihood Ratio Tests indicate high probability of evolution under positive selection, while in the case of *rrgB* the two tests are discordant.

**Figure 2:**
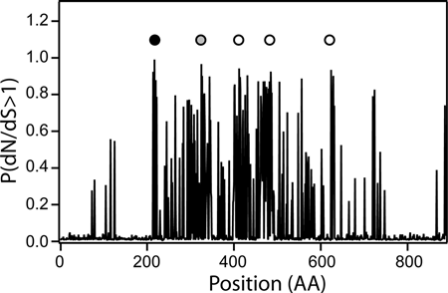
Positive selection on *rrgA*. Probability P(*d_N_/d_S_*>1) that a codon is under positive selection in *rrgA*. The dark circle marks a site for which both test I and test II indicate positive selection with probability greater than 99%; the light gray circle marks a site for which test I and test II indicate positive selection with a probability exceeding 95%; empty circles indicate sites under positive selection with probability exceeding 95% only according to test II.

For *rrgA*, which codes for the component that confers adherence to the pilus [1], [2], [4], both tests report a significantly higher likelihood of models incorporating positive selection over models of nearly neutral evolution. In Fig. 2 we report a plot of the probability P(*d_N_/d_S_*>1) that a codon has *d_N_/d_S_*>1 for *rrgA*, and sites for which the Bayes-Empirical-Bayes inference (BEB) method [26] (see Materials and Methods section) supports evolution under positive selection are evidenced. There are 5 amino acids that display significant evidence of evolution under positive selection. Although these sites are not in direct contact along the sequence, they could constitute a conformational epitope in the folded protein. Since in the presence of recombination statistical tests for positive selection relying on an estimated genealogy can give a high rate of false positives [27], we also analyzed *rrgA* using a Bayesian method which is able to identify sites under positive selection also in the presence of recombination [28], finding good agreement with the BEB analysis (see Text S1 and Fig. S1).

In order to verify if selection acts differently in the three evolutionary clades of *rrgA* (see Text S1 and Fig. S2), we have tested separately each clade by performing a likelihood-ratio test of the branch-site model implemented in *codeml*. The results are reported in Table 3. For clades I and III we did not find evidence of clade-specific positive selection. This is probably due to the short evolutionary distance separating these two clades (Fig. S2), which makes unlikely the fixation by positive selection of mutations specific to one clade with respect to the other. Instead in clade II, which is more distantly related to the other two, we found evidence of clade-specific positively selected codons, suggesting that the selective pressure caused by the host immune response might act differently on this pilus variant, possibly due to yet unknown clade-specific structural features.

**Table 3:** Test of positive selection of the *rrgA* gene.

Clade	LnL(freeω_2_)	LnL(ω_2_ = 1)	ΔL	χ^2^._1_ΔL)
I	−5243.64	−5244.15	0.51	0.31
II	−5214.10	−5246.46	32.35	**8.69E-16**
III	−5246.10	−5247.02	0.92	0.18

In this table we show the results of the log-Likelihood test of positive selection using the branch-site model A of PAML [24]. For each branch, the null model is model A with the constraint ω_2_ = 1. Also reported are the values of the χ^2^_1_ statistics. P-values below 0.01 are marked in bold. The test found evidences of positive selection for clade II, but not for clade I and III.

**Figure 3:**
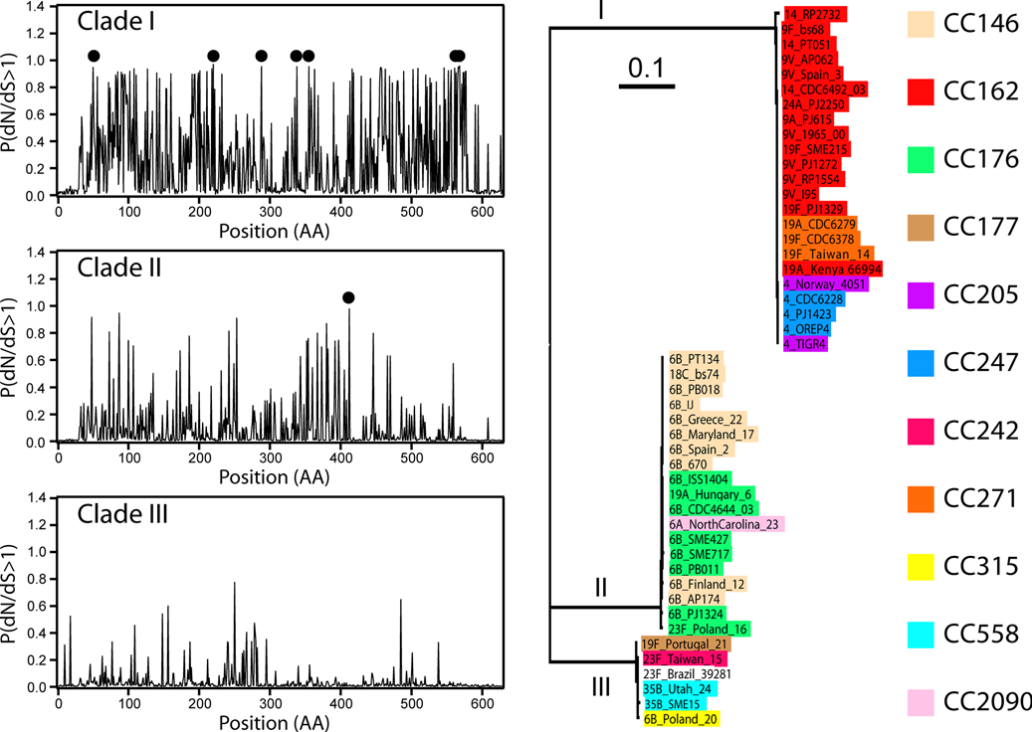
Positive selection on rrgB. Probability P(*d_N_/d_S_*>1) that a codon is under positive selection in *rrgB*. The branch-site model was tested separately for the three clades, marked on the phylogenetic tree on the right. From top to bottom: clade I, clade II clade III. The background color of the strain names identifies their MLST classification into clonal complexes. The likelihood ratio test supports the positive selection model only for clades I and II. Sites marked by dark circles are under positive selection with probability exceeding 99%.

In the case of *rrgB*, the pilus backbone[1], the M8-M7 likelihood ratio test indicated positive selection, while the M2a-M1a likelihood ratio test did not find evidence of positive selection. In order to assess if selection acts differently along the three major evolutionary clades of *rrgB* (see Fig. 3 and Fig. S2), we tested separately each clade by performing a likelihood-ratio test of the branch-site model implemented in *codeml*, similarly to what done for *rrgA*. The results are reported in Table 4. We found significant evidences of positive selection for both clade I and II, while there is less evidence for clade III. The resulting probabilities P(*d_N_/d_S_*>1) for each codon for the three clades are shown in Fig. 3, where sites under positive selection according to the BEB analysis are also evidenced. In clade III, we found no site passing the 99% significance cut-off for positive selection, although there are codons with relatively high values of the probability P(*d_N_/d_S_*>1) of having *d_N_/d_S_* greater than one. However, likelihood tests of positive selection are known to be conservative and BEB analysis often do not reach high values in branch-site models, and therefore we cannot exclude that also for clade III there are sites subject to positive selection which cannot be identified due to the relatively low sensitivity of the method.

**Table 4:** Test of positive selection of the *rrgB* gene.

Clade	LnL(freeω_2_)	LnL(ω_2_ = 1)	ΔL	χ^2^._1_ΔL)
I	−5898.49	−5904.65	6.17	**4.46E-04**
II	−5922.86	−5929.20	6.35	**3.67E-04**
III	−5934.21	−5936.92	2.71	0.02

In this table we show the results of the log-Likelihood test of positive selection using the branch-site model A of PAML [24]. For each branch, the null model is model A with the constraint ω_2_ = 1. Also reported are the values of the χ^2^_1_ statistics. P-values below 0.01 are marked in bold. The test found evidences of positive selection for clade I and clade II, but not for clade III.

### Balancing selection

**Figure 4:**
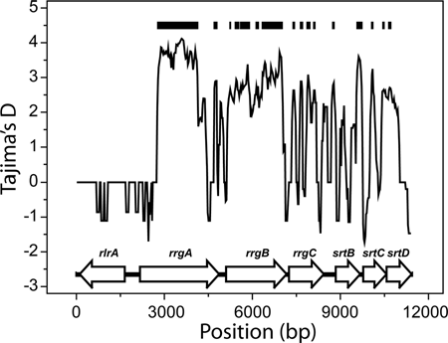
Tajima's D test of neutral evolution. Tajima D computed on a sliding window of length 100 bp shifted by 25 bp at each step. Dark lines mark the regions where the value of D is positive with p-value<0.001. Also shown are the positions of the ORFs along the sequence. Most of the regions with significantly positive values of D are located in the regions encoding for the RrgA and RrgB proteins.

As reported above, in the molecular phylogenesis of the *rrgA* and *rrgB* genes we can identify three distinct clades (Fig. S2), which coexist in the circulating strains. To assess if this population structure is consistent with a neutral model of evolution, we have computed Tajima's D [29] along the *rlrA* sequence, averaged over a sliding window of 100 bp. The results are plotted in Fig. 4 as a function of the position along the sequence. Dark lines show the regions where D is different from zero with p-value below 0.001. These regions are mainly included in the ORFs encoding the *rrgA* and *rrgB* genes, while the value of D drops markedly in the region flanked by the *rrgA* and *rrgB* genes. In the rest of the islet, D assumes values that, although positive in most cases, are not significantly different from 0. While D equal to 0 indicates an absence of population structure, a positive value of D can be attributed either to demographic effects, when two or more subpopulations of related individuals are sampled, or to the presence of balancing selection, when two or more clusters of haplotypes coexist in the same population [29]. In both cases there is an excess of pair-wise differences between individuals belonging to different groups. We found no correlation between the geographic origin, time of isolation or associated disease of the strains and their classification into one of the three clades. Moreover, since demographic effects act evenly across the entire genome, the fact that the significantly positive values of D are limited to the *rrgA* and *rrgB* genes indicates that the presence of distinct alleles of these genes in the population cannot be attributed only to the existence of independently evolving subpopulations.

### Recombination

**Figure 5:**
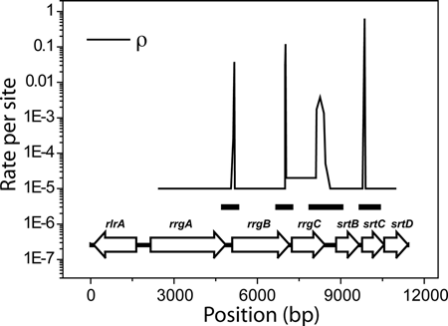
Recombination Rate. Plot of the recombination rate ρ per base pair along the sequence. Also shown are the positions of the ORFs. The recombination hotspots, represented by the peaks in ρ, are closely associated with the intergenic regions. Due to the low level of sequence divergence, ρ is not computed in the region encoding for the *rlrA* transcriptional regulator. The dark intervals mark the positions of 4 recombination hotspots predicted using sequenceLDhot, which are in good agreement with the positions of the peaks of ρ.

A molecular phylogenetic analysis of the different genes in the *rlrA* islet shows that these genes have incompatible genealogies (Fig. S2), suggesting that the *rlrA* islet has undergone several recombination events. In Fig. 5 we show a plot of the local recombination rate ρ obtained using an extended composite likelihood method [12], [30]. The positions of the ORFs encoded in the *rlrA* islet are also shown. There are four sharp peaks in ρ which either coincide with or are very close to the intergenic regions, *i.e.* non-coding regions flanked by two adjacent genes. In order to confirm that these peaks correspond to recombination hotspots, we used an independent method that is able to identify regions where the recombination rate is much higher than in the neighboring regions [13]. The positions of the predicted hotspots are also shown in Fig. 5 as dark intervals, and they agree with the positions of the peaks in the value of ρ.

The *rrgC* ORF has a slightly higher background recombination rate than the other ORFs. The higher level of recombination in this region is also confirmed by the larger size of the predicted hotspot centered between the *rrgC* and *srtB* ORFs (see Fig. 5), which partially overlaps the *rrgC* gene. Since, as recently shown by computer simulations [31], recombination can act as a homogenizing mechanism by the random exchange of short DNA sequences, the higher value of ρ in this region could contribute to explain the remarkably low level of sequence variability of *rrgC* compared to the other two structural components *rrgA* and *rrgB* (see Table 1).

**Figure 6:**
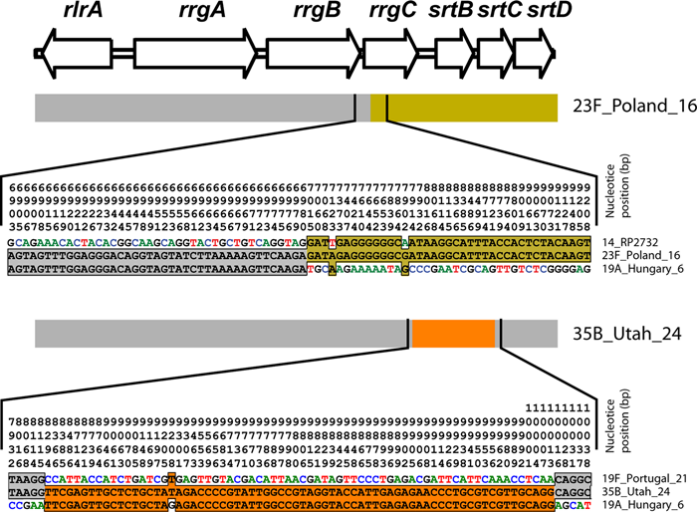
Detection of recent recombination events. Example of two recent recombination events that can be detected by comparing triples of sequences. Only the polymorphic sites are shown, and coordinates are reported vertically above each site. In both cases the insertion of a recombinant sequence (marked in color) in a non-recombinant background (marked in grey) is identified by a switch in the pattern of sequence conservation at the polymorphic sites.

**Figure 7:**
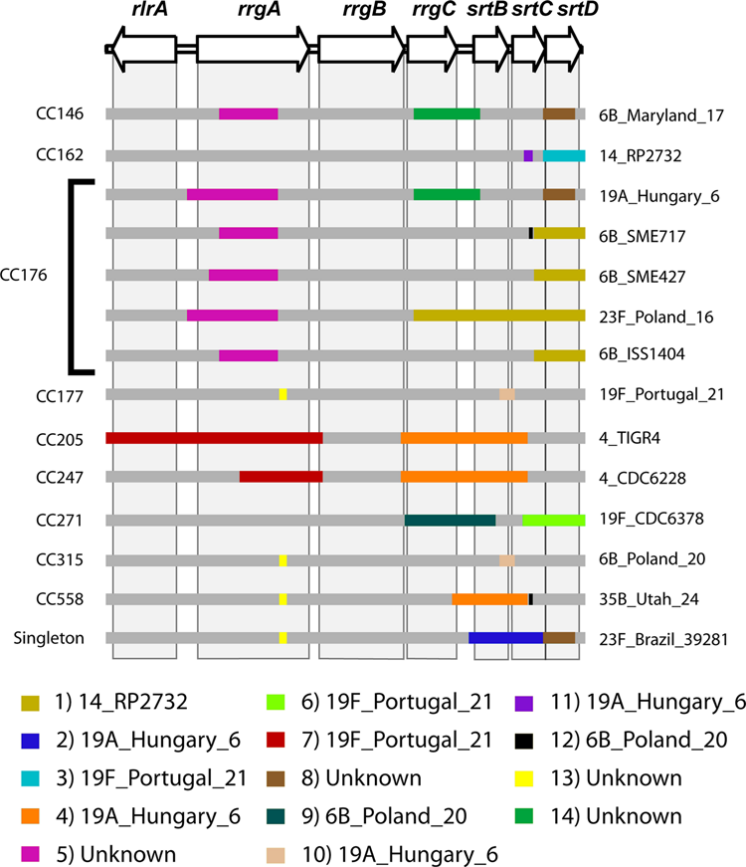
Schema of recent recombination events. Schematic representation of the main recent recombination events occurred within a representative subset of the sequenced strains. Each line represents a recombinant sequence, and color boxes indicate the insertion of a foreign sequence. Colors represent each unique insertion, and the name of the putative originating strain is reported in the color legend. Gray shaded areas mark the position of the ORFs on the recombinant sequences.

Recombination events can be detected by visual inspection of sequence data from an abrupt switch in the similarity relationships amongst a triple of sequences, as shown in Fig. 6. However, these evidences decay rapidly with time due to the evolution of the recombining sequences, allowing the direct identification of only relatively recent events. In Fig. 7 we show a schematic representation of the most recent recombination events which can be identified in our panel of strains using a wide range of comparative methods [32] (see also Text S1 and Table S2). Each line represents one strain, and the insertions are color coded according to the most likely origin of the inserted sequence. Boxes overlapping the same region and sharing the same color in different strains have probably originated by a single acquisition in a common ancestor. With the exception of CC176, only one strain per CC is shown in Fig. 7, because all strains belonging to the same CC display evidences of the same insertions, indicating that most recombination events predate the formation of MLST clonal complexes. In CC176 the sequences inserted in event 1 and 5 have different borders and lengths in the different isolates, indicating that CC176 is an ancient complex, and that the evidences of these insertions in the different strains have been overwritten by more recent evolution. This feature is also shared by two other insertions, namely event 4 and 7, where the recombinant sequences belong to CC205, CC247 and CC558, and to CC205 and CC247, respectively. However, differently from events 1 and 5, in these cases the insertions, being shared by strains belonging to different CCs, occurred probably in a common ancestor of the founders of these CCs. One of the CC176 strains, namely Hungary 19A, has a pattern of insertions different from the other CC176 strains, but very similar to the CC146 strains, suggesting for this strain a genealogy that violates the MLST schema, or a recent acquisition of the entire locus from a CC146 strain.

In many cases the association of the borders of the insertions with the intergenic regions is evident. Gene mosaicism is mainly confined to the *srtB*, *srtC*, and *srtD* genes. These genes, together with *rrgC*, appear to have been frequently exchanged across the circulating *S. pneumoniae* strains. Since recombination is a powerful force limiting the genetic drift amongst isolates [31], this finding is consistent with the fact that these ORFs are the ones with the lowest level of diversification. At the other end of the spectrum *rrgB*, which is the most variable gene, displays no evidence of recent recombination events, while *rrgA*, which is the second most variable gene, displays evidence of two large and one small insertions, one of which, (event 5) probably originated outside the panel of strains that we sequenced.

## Discussion

The *rlrA* islet of *S. pneumoniae* codes for elongated structures, or pili, protruding outside the bacterial cell, enhancing the ability of the bacterium to adhere to epithelial cells. Previous studies have shown that this islet is present in approximately 30% of the *S. pneumoniae* isolates, and that the distribution of this islet correlates well with the Clonal Complexes defined by MLST, which are nearly always composed either by pilus-positive or pilus-negative strains [6].

Our comparative analysis of the *rlrA* islet sequenced in a representative set of clinical isolates shows that the mechanism of evolution of each gene in this locus is correlated with its function in the synthesis and assembly of pili. In the region encoding for the *rrgA* and *rrgB* proteins, which are two of the three main structural components of the pilus, the evolution is driven by the selection of new escape mutants by the interaction with the host through a mechanism of positive selection. Phylogenetic analysis of the *rrgA* and *rrgB* genes identifies three distinct evolutionary clades connected by long internal branches, and population genetics analysis evidences that the variability in this region is incompatible with a single homogeneous population. In the classical model of diversifying selection, this kind of variability in proteins with antigenic properties is attributed to the selective advantage of strains bearing low frequency alleles, due to the higher number of susceptible hosts [22], [33]. This negative frequency-dependent selection prevents a single allele from fixating by random drift as expected under neutral evolution. However, doubts about the general applicability of this model in the case of *S. pneumoniae* have recently been risen [34], suggesting that the role of host immunity in determining the population variability of antigens could be less important than previously assumed. Alternative explanations of the data, including, for instance, adaptation of the different alleles to different kinds of hosts are possible and require further epidemiological and functional studies.

For the other genes forming the *rlrA* islet, which encode proteins that are less exposed to the host immune system, recombination is the main mechanism of diversification, as previously reported for *S. pneumoniae* [11]. However, a map of the recombination rate along the sequence of the *rlrA* islet shows that crossover breakpoints are not distributed randomly along the sequence, and identifies four recombination hotspots that are located very close to the (*rrgA*,*rrgB*), (*rrgB*,*rrgC*), (*rrgC*,*srtB*), and (*srtB*,*srtC*) intergenic regions. This finding is reminiscent of the correlation between recombination hotspots and non-coding regions in the human genome [12]. Visual inspection of the most recent recombination events occurred in this region shows that while the sequence of the *rrgA*, *srtB* and *srtC* genes contain evidence of recent crossover events, the *rrgB*, *rrgC* and *srtD* genes have evolved mainly through the accumulation of mutations, and have been exchanged amongst the different strains only as discrete units. As a whole, the region of the genomic island encoding the *rrgC*, *srtB*, *srtC* and *srtD* genes displays a high level of mosaicism, with recombination probably contributing to decrease the fixation time of beneficial mutations [35], thus reducing the probability of severe evolutive bottlenecks. A recent example of this mechanism in a different context is the capsular switch under the pressure of vaccination which has been reported in *S. pneumoniae* [36], where the capsular locus of a non-vaccine serotype has been acquired by a virulent strain of a vaccine serotype.

These findings contribute to elucidate the adaptation of organelles involved in host-pathogen interaction to the host immune response, and are relevant to understand how the populations of pathogenic bacteria react to the changes in their ecological niche. A better characterization of these phenomena and of the relevant timescales could help to clarify the population structure of pathogenic bacteria and to predict the changes caused by the introduction of large-scale vaccination.

## Materials and Methods

### Strain selection

A worldwide collection of 424 strains of *S. pneumoniae* including 70 serotypes and both carriage and invasive strains had previously been screened for the presence of the *rlrA* islet [6]. Of the 130 strains that tested positive, 44 isolates that are representative of the main MLST Sequence Types (STs), serotypes and geographic locations were selected. The list of sequenced strains is reported in Table S1. Additionally, the genetic sequence encoding for the *rlrA* islet were extracted from the genomes of the strains SP9-BS68 and SP18-BS74 downloaded from the NCBI Web site (http://www.ncbi.nlm.nih.gov), and from the genomes of strains TIGR4 and 670 downloaded from the TIGR web site (http://www.tigr.org).

### rlrA islet sequencing

TIGR4 [37] and 670 *rlrA* nucleotide sequences were analysed and a set of 22 oligonucleotides matching in homologous regions inside the islands was designed. Sequences were obtained by use of an ABI 3730xl DNA Analyzer and assembled with Vector NTI 9.1.

Multi Locus Sequence Typing (MLST). MLST was performed as previously described [38]. Briefly, internal fragments of the *aroE*, *gdh*, *gki*, *recP*, *spi*, *xpt* and *ddl* genes were amplified by PCR directly on the bacteria using the primers pairs indicated at http://spneumoniae.mlst.net/misc/info.asp#experimental. Sequences were obtained by use of an ABI 3730xl DNA Analyzer. Alleles from the MLST website (http://spneumoniae.mlst.net) were downloaded for convenient alignment analyses and Sequence Type (ST) determination. New allelic profiles were submitted to the MLST database for ST assignment.

*Clonal Complex (CC) determination*. CCs are groups of STs sharing a recent common ancestor. These may be defined using the eBURST program, which also identifies the ancestral ST [7]. To explore the relationship between pilus presence in our dataset and CC, we ran eBURST with default settings on the entire MLST database and subsequently, on the basis of this analysis, assigned each ST within our dataset to a clonal complex. In this work we named CCs according to the ST number of the eBURST predicted founder, the latter defined as being the ST with the greatest number of single-locus variants within the CC.

### Sequence alignments

Coding and non-coding sequences were aligned separately using T-coffee 5.05 [39]. For coding sequences, the amino-acidic sequences were aligned and then the resulting alignments were back translated into multiple alignments of nucleotide sequences using the T-coffee reformat procedure. The alignment of the entire *rlrA* islet was reconstructed by chaining the alignments of the protein coding genes and of the intergenic regions in the order in which they are present in the islet.

### Measurements of sequence diversity and of Tajima D

The per-site mutation rate in a set of sequences can be estimated from the average number π of differences between pairs of sequences, or from the number of segregating sites (Watterson's estimator θ). The normalized difference of these two estimators, known as Tajima's D, can be used to identify violations from a neutral coalescent model of evolution. If sequences evolve according to a neutral coalescent model, the two estimates of the mutation rate coincide and D = 0. Instead, a negative value of D is obtained in the case of a star-shaped genealogy, as obtained, for instance, during recovery after a selective sweep that fixed a favored allele and temporarily decreased the amount of polymorphism at nearby sites. A positive value of D is obtained instead when the phylogenetic tree describing the evolution of a set of sequences has a deep split into two or more groups. In other words, D is negative when most coalescent events in a group of sequences occur near the recent times, while the time to most recent common ancestor of all sequences is longer than expected. From the point of view of the selective forces driving evolution, a significantly positive value of D is compatible with a balancing selection preventing a single allele from being fixed in the population and maintaining an higher than expected level of diversity. The nucleotide diversity π with Jukes-Cantor correction [40], the Watterson's estimator of the population mutation rate per site θ [41] and Tajima D [29] were computed using DnaSP 4.10 [42], using a sliding window of length 100 bp shifted by 25 bp at each step.

### Phylogenetic reconstruction

Gene trees were computed using MEGA v.4 [43] using the Neighbor Joining algorithm from distance matrices between DNA sequences computed using the Maximum Composite Likelihood Method [44].

### Detection of positive selection

The test for positive selection was conducted using a likelihood-based method, where the likelihood of different evolutionary models is computed given a gene sequence alignment and a reconstruction of the phylogenetic history of the genes. Since in some extreme cases recombination can affect the identification of sites experiencing positive selection using phylogenetic methods, *rrgA* was subsequently tested using a Bayesian method that does not rely on a given evolutionary history and is able to estimate the presence of positive selection also in the presence of recombination.

i) Likelihood method. For each coding sequence, we used the *codeml* program of the PAML 3.15 [24] suite to conduct a likelihood ratio test between different evolutionary models. We tested the M0, M1a, M2a, M7 and M8 site models for one ratio, nearly neutral, positive selection, beta distributed rates and beta distributed rates+positive selection, respectively. We then determined the most likely model of evolution using the likelihood ratio test for the pairs M0–M1a, M0–M2a, M1a–M2a, M0–M7, M0–M8 and M7–M8. For those cases supporting positive selection, the sites under selective pressure were identified from a high posterior probability to belong to the classes with *d_N_/d_S_*>1. Computing this probability requires the averaging over the distribution of model parameters and is therefore computationally demanding. Reliable numerical estimates of this probability were obtained by using the Bayes Empirical Bayes (BEB) technique [26], that accommodates uncertainties in the *d_N_/d_S_* distribution by integrating over a prior of the parameters, while the branch lengths, which are known to have a minor impact on the inference concerning *d_N_/d_S_*, are left fixed at their maximum likelihood estimate.
  For the three major clades of *rrgA* and *rrgB* (see Fig. 3 and Fig. S2), we also tested the branch-site model A in *codeml*, where a foreground clade is allowed to have branch-specific and site-specific positive selection. For each clade, we contrasted the positive selection model with the same model with ω_2_ = 1.
ii) Bayesian method. We used a method based on a coalescent with recombination process, implemented in the omegaMap program v.0.5 [28] to determine the position of the codons that are likely to evolve under positive selection. By explicitly modeling recombination, this method has a low false positive rate in the presence of recombination and provides robust inference on the variation of the *d_N_/d_S_* ratio due to selection.

### Detection of recombination events

The recombination events were detected using the RDP 3.14 suite [32] with default options, requesting that the putative recombination event was detected by at least 3 different methods with P-value<0.05.

### Recombination rate plot

The local recombination rate has been estimated using the program LDhat included in the RDP v3.14 suite [32] using the gene conversion model and an average tract length of 100 bp. In this method, a recombination map composed by a series of intervals each with constant recombination rate is fit on the multiple alignment. For each interval, the recombination rate is estimated by using an extended composite likelihood approximation of the coalescent likelihood [12], [30]. In this approximation, the likelihood of the observed set of polymorphisms for each segment with a local value of the recombination rate is estimated by importance sampling over the distribution of the ancestral recombination graphs [45]. This method, by explicitly modeling the evolution of each sequence segment in the presence of recombination and mutation, provides estimates of the recombination rates that are not artificially inflated by the presence of related sequences in the dataset. To avoid over-fitting, smoothness of the resulting rates is encouraged by the adoption of an average length for the recombining sequences. Increasing the adopted average tract length of 100 bp detects less putative recombination events by missing many of the smaller sequence inserts, while further decreasing this value does not change the results.

### Detection of recombination hotspots

The presence of recombination hotspots inferred from the recombination rate plot was confirmed using the program sequenceLDhot [13] using the an average mutation rate per base pair of 0.03, an average background recombination rate per base pair of 10^−5^, and average hotspot width of 300 bp. Candidate hotspots with p-value<10^−3^ were selected. The position of the hotspots was estimated by the extended hotspot region approach [13] with LRm = 4.

## Supporting Information

Text S1 (0.03 MB DOC)

Figure S1 Positive selection on *rrgA*. Here we show the probability P(*d_N_/d_S_*>1) that a codon is under positive selection in *rrgA* using PAML and omegaMap.
(6.12 MB TIF)

Figure S2 Phylogenetic Trees of the *rlrA* islet genes. Here we show the Neighbor Joining phylogenetic trees of *rrgA*, *rrgB*, *rrgC*, *srtB*, *srtC*, *srtD* (left to right, top to bottom). Due to the high level of sequence conservation, the tree for the rlrA transcriptional regulator is not shown. The background colors of the strains names show their clonal complex determined by eBURST. Since rrgB is the most variable protein, the strains are classified into three groups corresponding to clade I, II and III indicated in the *rrgB* phylogenetic tree.
(26.75 MB TIF)

Table S1 Name, capsular serotype, MLST Sequence Type, eBURST Clonal Complex, and country of isolation of the strains used for this study.
(0.01 MB XLS)

Table S2 Details of the recombination events detected in the sequence dataset using the RDP program suite.
(0.06 MB XLS)
