# Supplementary material for: Pilus Operon Evolution in Streptococcus pneumoniae Is Driven by Positive Selection and Recombination
Source: PLoS One. 2008 Nov 6;3(11):e3660. doi: 10.1371/journal.pone.0003660 (PMC2576445; doi:10.1371/journal.pone.0003660)
Supplement: Text S1 — (0.03 MB DOC) [file pone.0003660.s001.doc]

**Supporting information.**

**Positive selection.** In the presence of recombination statistical tests for positive selection relying on an estimated genealogy can give a high rate of false positives[1]. In order to confirm the results obtained with PAML [2], we analyzed *rrgA* using the program omegaMap v0.5, that implements a Bayesian method which is able to identify sites under positive selection also in the presence of recombination[3]. In Fig. S1 we report the probability that a codon in *rrgA* evolves under positive selection P(dN/dS>1) obtained both using PAML using the phylogenetic tree reported in Fig. S1 (black line) and the same quantity obtained using omegaMap. The two quantities are in good agreement, predicting evolution under positive selection for the same regions.

**Phylogenetic analysis.** In Figure S2 we show the Neighbor Joining phylogenetic trees of the genes forming the *rlrA* islet (from top to bottom, left to right, *rrgA*, *rrgB,* *rrgC*, *srtB*, *srtC*, and *srtD*). The background colors of the strain names indicate the MLST Clonal Complexes (CC) to which the strains belong. Strains belonging to the same CC tend to be very close in each tree, suggesting that MLST describes accurately the recent evolution of this locus. The phylogenetic trees *rrgA* and *rrgB*, which are located next to each other in the *rlrA* islet, share the same general structure partitioned in three major clades, with the only exception of the clade switch of two serotype 4 clonal complexes CC205 and CC247. This partitioning is also shared by the phylogenetic tree based on the nucleotic sequence of the intergenic region enclosed by the *rrgA* and *rrgB* genes (data not shown), suggesting that recombination has played only a marginal role in the evolution of the portion of the *rlrA* islet containing the *rrgA* and *rrgB* genes. Instead, the structure of the trees for *rrgC*, *srtB*, *srtC* and *srtD* varies widely, indicating a higher incidence of recombination in the region encoding for these genes. Also the branch length of these trees is much smaller than in the case of *rrgA* and *rrgB*, showing that the *rlrA* islet contains regions with very different sequence diversity. According to their position in the *rrgB* tree, the strains will be classified into clade I, II, and III as indicated in Figure S2.

**Strain panel.** The strains were selected to give a good coverage of the population of circulating strains of *S.pneumoniae*. In Table S1 we report, for each strain, capsular Serotype, MLST Sequence Type, MLST Clonal Complex obtained using eBURST [4], the pilus clade classification, the associated disease (where known) and the geographic locus of isolation.

**Recombination events.** We have analyzed the nucleotidic multiple alignments of the *rlrA* islet using the RDP[5] program suite with default options, requesting that the putative recombination event was detected by at least 3 different methods with P-value<0.05. In Table S2 we report the details of the results of this analysis. For each putative recombination event we report the breakpoints position in the multiple alignment, the id of the recombinant sequence, the id of the minor and major parents, and the event e-scores for each of the detection methods used.

**Literature Cited**

1. Anisimova M, Nielsen R, Yang Z (2003) Effect of recombination on the accuracy of the likelihood method for detecting positive selection at amino acid sites. Genetics 164: 1229-1236.

2. Yang Z (2007) PAML 4: phylogenetic analysis by maximum likelihood. Mol Biol Evol 24: 1586-1591.

3. Wilson DJ, McVean G (2006) Estimating diversifying selection and functional constraint in the presence of recombination. Genetics 172: 1411-1425.

4. Feil EJ, Li BC, Aanensen DM, Hanage WP, Spratt BG (2004) eBURST: inferring patterns of evolutionary descent among clusters of related bacterial genotypes from multilocus sequence typing data. J Bacteriol 186: 1518-1530.

5. Martin DP, Williamson C, Posada D (2005) RDP2: recombination detection and analysis from sequence alignments. Bioinformatics 21: 260-262.
