# Supplementary figures and images for: Pilus Operon Evolution in Streptococcus pneumoniae Is Driven by Positive Selection and Recombination
Source: PLoS One. 2008 Nov 6;3(11):e3660. doi: 10.1371/journal.pone.0003660 (PMC2576445; doi:10.1371/journal.pone.0003660)

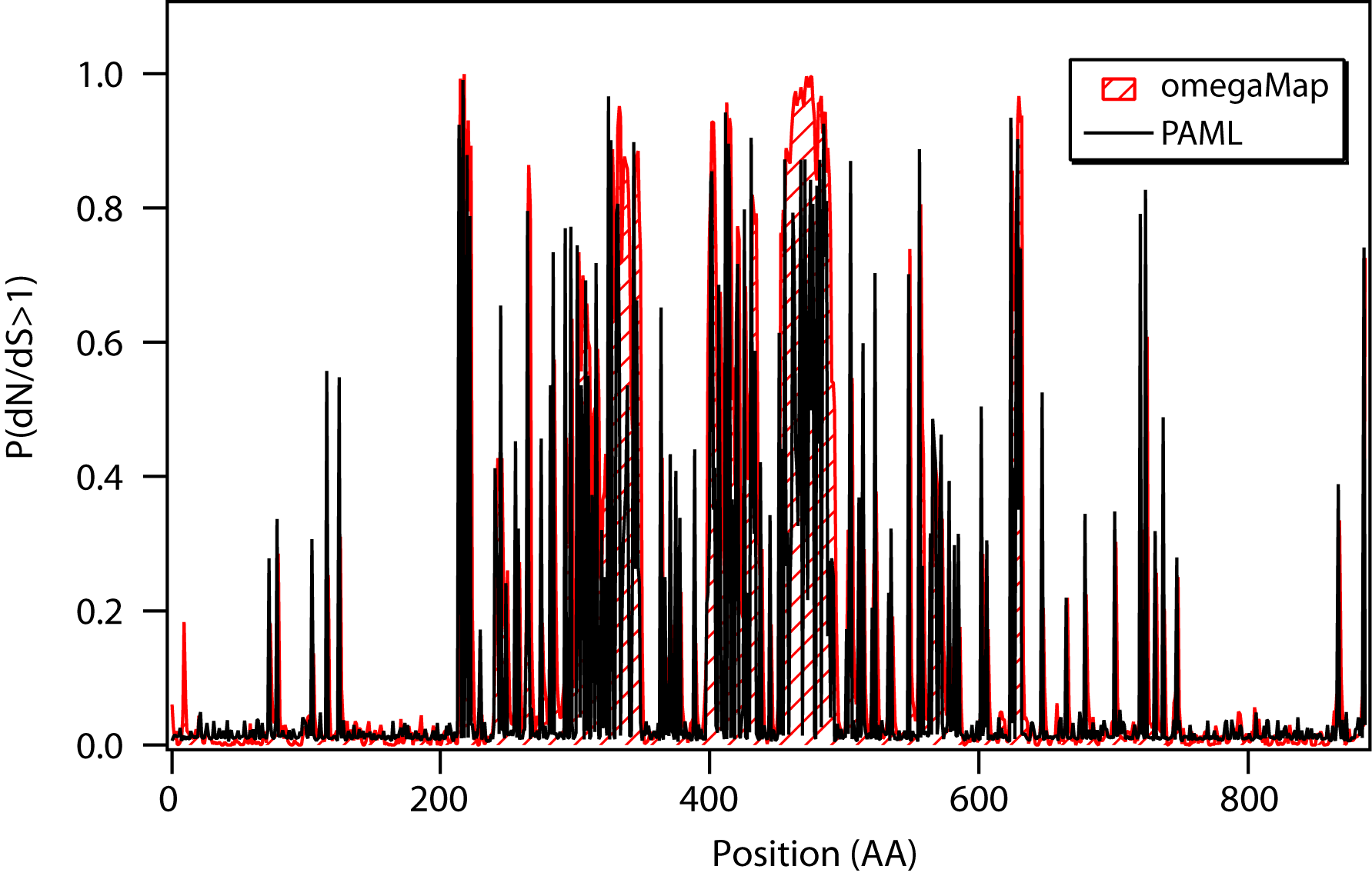

Supplement: Figure S1 — Positive selection on rrgA. Here we show the probability P(dN/dS>1) that a codon is under positive selection in rrgA using PAML and omegaMap. (6.12 MB TIF) [file pone.0003660.s002.tif]

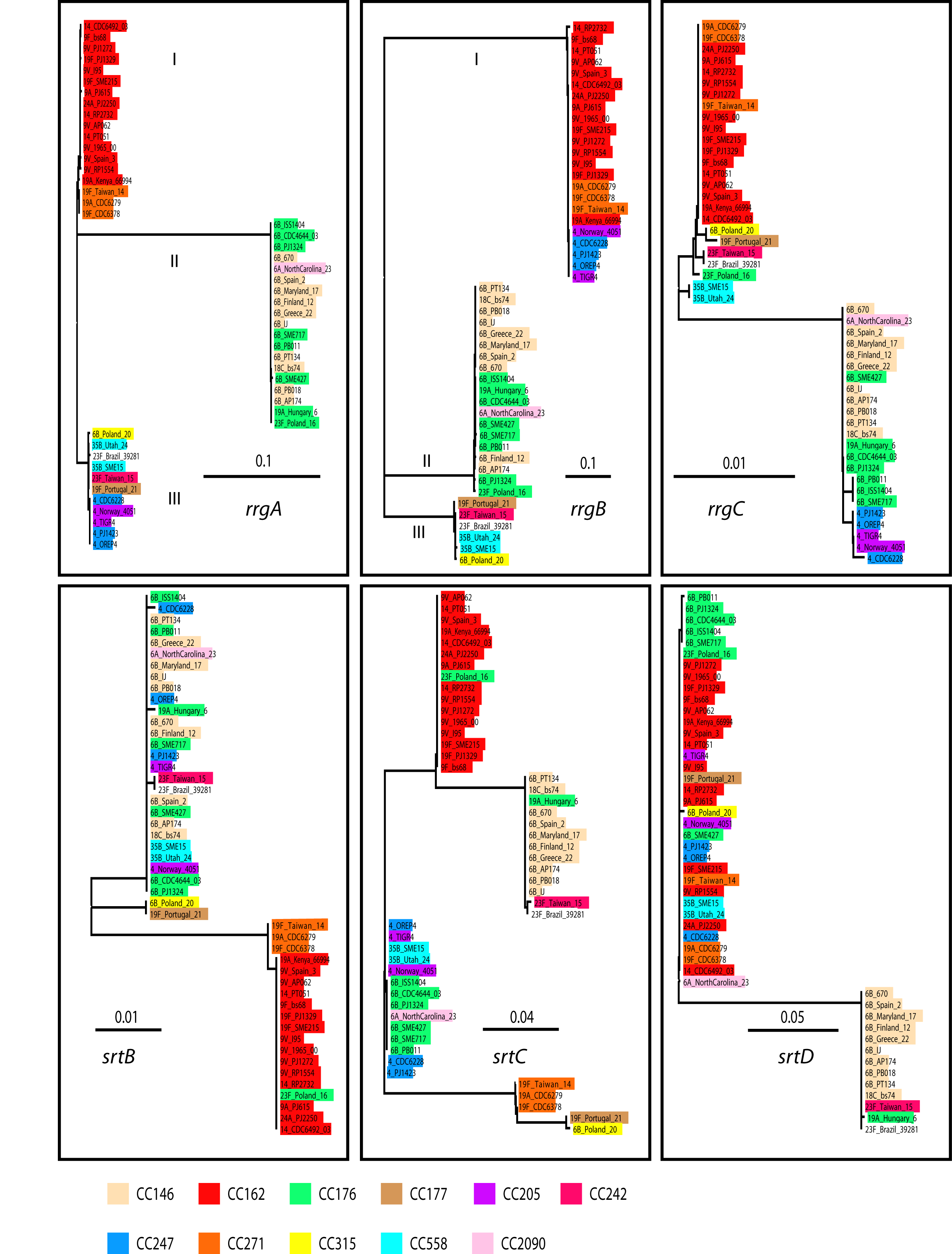

Supplement: Figure S2 — Phylogenetic Trees of the rlrA islet genes. Here we show the Neighbor Joining phylogenetic trees of rrgA, rrgB, rrgC, srtB, srtC, srtD (left to right, top to bottom). Due to the high level of sequence conservation, the tree for the rlrA transcriptional regulator is not shown. The background colors of the strains names show their clonal complex determined by eBURST. Since rrgB is the most variable protein, the strains are classified into three groups corresponding to clade I, II and III indicated in the rrgB phylogenetic tree. (26.75 MB TIF) [file pone.0003660.s003.tif]
